# Semi-automated seizure detection using interpretable machine learning models

**DOI:** 10.21203/rs.3.rs-4361048/v1

**Published:** 2024-05-30

**Authors:** Pantelis Antonoudiou, Trina Basu, Jamie Maguire

**Affiliations:** Tufts University School of Medicine; Tufts University School of Medicine; Tufts University School of Medicine

## Abstract

Despite the vast number of seizure detection publications there are no validated open-source tools for automating seizure detection based on electrographic recordings. Researchers instead rely on manual curation of seizure detection that is highly laborious, inefficient, error prone, and heavily biased. Here we developed an open-source software called SeizyML that uses sensitive machine learning models coupled with manual validation of detected events reducing bias and promoting efficient and accurate detection of electrographic seizures. We compared the validity of four interpretable machine learning models (decision tree, gaussian naïve bayes, passive aggressive classifier, and stochastic gradient descent classifier) on an extensive electrographic seizure dataset that we collected from chronically epileptic mice. We find that the gaussian naïve bayes and stochastic gradient descent models achieved the highest precision and f1 scores, while also detecting all seizures in our mouse dataset and only require a small amount of data to train the model and achieve good performance. Further, we demonstrate the utility of this approach to detect electrographic seizures in a human EEG dataset. This approach has the potential to be a transformative research tool overcoming the analysis bottleneck that slows research progress.

## Introduction

Epilepsy is a devastating neurological disorder characterized by recurrent seizures. Detection of seizures by evaluation of the electroencephalogram (EEG) is critical for diagnosis and Epilepsy research (Flink et al., 2002). However, manual detection, which is the current standard of practice, is laborious, error prone, requires extensive training and expertise, and results in large variability (Diachenko et al., 2022). Artificial intelligence and Machine learning (ML) techniques hold great promise in assisting clinical diagnosis and transforming research (Rajpurkar et al., 2022; Yu et al., 2018); as can be seen by the prompt FDA approval of AI tools (Benjamens et al., 2020).

A wealth of ML methods have been proposed for automating the task of seizure detection (Shoeibi et al., 2021; Siddiqui et al., 2020). Specifically, deep learning techniques automate feature extraction and perform remarkably well in seizure detection among other tasks (Cho & Jang, 2020; Rajpurkar et al., 2022; Shankar et al., 2022; Shoeibi et al., 2021; Yuan et al., 2019). However, deep learning techniques are not easily interpretable (Rudin, 2019). This is an important issue as many clinicians and scientists are reluctant to employ these methodologies due to lack of trust (Pinto et al., 2022; Rudin, 2019); as the characterization of parameters that constitute a seizure is not clear. Indeed, even high-performing deep learning models can be deceived by small modifications to an image (Su et al., 2019) or may erroneously identify images as objects that are unrecognizable to humans (Nguyen et al., 2015). Furthermore, deep learning models often require a large amount of training data to reach good performance levels and can be prohibitive to train due to expensive computational resources (Rajpurkar et al., 2022; Yu et al., 2018). Finally, these existing approaches make it difficult to extract the electrographic features which may be informative regarding seizure generation and progression.

Here we have employed widely-used and interpretable ML models from the scikit-learn python library (Pedregosa et al., 2011) and tested their performance on detecting seizures from a well-established mouse model of epilepsy. We found that these interpretable models with combined extraction of simple features were sufficient to detect all seizures in our preclinical dataset as well as electrographic seizures in a human EEG dataset. To make these pipelines accessible to the Scientific community we created an open-source python application (SeizyML) to couple high model sensitivity with manual verification of the detected seizures. This semi-automated approach significantly reduces time required for seizure detection while providing high sensitivity and accuracy.

## Methods

### EEG/LFP Dataset

The data used here for training, validation, and testing of the models were obtained from recordings collected in our previous study (Basu et al., 2022), where all methods were performed in accordance with the relevant guidelines and regulations, approved by the Tufts University Institutional Animal Care and Use Committee, and in accordance with the ARRIVE guidelines. Briefly, adult mice were injected with kainic acid in the ventral hippocampus (vHPC) and were then implanted with a stainless-steel wire in the vHPC and a stainless-steel screw fixed above frontal cortex (FC). Data were acquired at 4000 samples per second. The data were split into training (11 mice, 4224 hours), and testing (15 mice, 5511 hours) datasets. Subsequently data were divided in 5 second windows and downsampled to 100 Hz with an antialiasing filter, as this has been shown to achieve excellent performance (Cho & Jang, 2020; Jang & Cho, 2019). Data were also high-pass filtered at 2 Hz to remove baseline drift and extreme outliers (> 25 standard deviations) and were replaced with the median of each 5 second window. Then 9 features were extracted for each channel (autocorrelation, line length, root mean square, mean absolute deviation, variance, standard deviation, power (2–40Hz), energy, envelope amplitude) and 3 features were extracted for cross channel metrics (cross-correlation, covariance, absolute covariance) (Table 1) that resulted in a total of 21 features (9 for each channel + 3 cross channel). The features were converted into z-scores to equalize their contribution to the machine learning models.

### Feature Selection

In order to remove redundant information and select the most meaningful features we first removed highly correlated features with Pearson coefficient *r* > 0.99, which resulted in 12 features that constituted the first feature-set (Table 2, Column 1). We then selected the 4 and 8 best ranking features from ANOVA comparison with target variable (Table 2, Column 2&3) and from mutual information (Table 2, Column 4&5), resulting in a total of 5 feature-sets (Table 2).

### Feature Contributions

Feature contributions to model predictions were obtained as follows:

1. For the DT model, we retrieved feature importance from the trained DT models (Sklearn *DecisionTreeClassifier*), where feature importance was calculated as the Gini importance.
2. For the SGD model, we calculated feature weight as the absolute weights for each feature from the trained SGD model (Sklearn *SGDClassfiier*).
3. For the GNB model, we calculated feature separation score based on extracted parameters of each class from the trained GNB model (Sklearn *GaussianNB*):

$$\text{FeatureseparationScore}=\left|\frac{\mu_{1}-\mu_{2}}{\sigma_{1}+\sigma_{2}}\right|$$

Where *μ* is the mean and σ is the standard deviation for each class.

*Feature contributions were normalized to have a sum of 1 for each model metric.

### Model Selection and Training

We selected four models for seizure detection, gaussian naïve bayes (GNB), decision tree (DT), stochastic gradient descent classifier (SGD) and passive aggressive classifier (PAC) from the scikit-learn (Sklearn) toolbox (Pedregosa et al., 2011). We selected models based on interpretability and their ability to train on large datasets. The SGD model was used with log/hinge loss to implement logistic regression/support vector machine models, respectively. This was done as logistic regression and support vector machine models could not train effectively on large datasets. The PAC model was selected as another implementation of a support vector machine model (hinge loss) with a different optimization algorithm (Crammer et al., 2006). The k-nearest neighbors model was not used as it was too slow and therefore not useful in practice during model predictions. The models were then tuned by performing grid-search using balanced accuracy as the fit metric to optimize their hyperparameters. Hyperparameter search was performed using 4-fold cross validation (75% training subset, 25% validation subset) of the training dataset (11 mice, 4224 hours). Sklearn’s *StratiedKfold* method was used to ensure that the class distributions were similar in training and validation datasets. The tuning was performed for each model, for every feature-set, and the resulting best hyperparameters were selected (Table 3). Each model/feature-set combination from Table 3 was then trained 5 times using *StratiedKfold* (5 folds, 80% training subset) and evaluated on the testing dataset (15 mice, 5511 hours).

### Human Dataset

To validate our models on a human EEG dataset with multiple channels we utilized the widely used Boston Children’s Hospital MIT dataset (CHB-MIT), that we obtained from PhysioNet (https://physionet.org/content/chbmit/1.0.0/), in accordance with relevant guidelines and regulations. The dataset consists of 24 scalp EEG recordings of 23 people with epilepsy (one subject was recorded twice). Subjects had an age range of 1.5–22 years and continuous recordings (segmented into multiple les per patient) were obtained after withdrawal of anti-seizure medication. Recordings were acquired at 256 samples per second and each recording had between 23–26 channels using the international 10–20 system configuration. For preprocessing, we high-pass filtered the data at 0.5 Hz, divided the data into 5 second bins, and joined all segmented les into one HDF5 le for each subject. We also created one accompanying CSV le for each subject containing ground truth data from each subject’s summary text le rounded to nearest 5 second bin. We selected the following 18 channels as they were common across patients (*FP1-F7, F7-T7, T7-P7, P7-O1, FP1-F3, F3-C3, C3-P3, P3-O1, FZ-CZ, CZ-PZ, FP2-F4, F4-C4, C4-P4, P4-O2, FP2-F8, F8-T8, T8-P8, P8-O2*) and only excluded 3 recordings from patient 12 as the recorded channels were different (chb12_27, chb12_28, chb12_29). For two out of the three classification types (See Human Model Training section: leave-one-out, and inter-subject) seizure segments were upsampled using a sliding window of 0.5 seconds in the training dataset.

### Human Feature Selection

The features extracted from the CHB-MIT dataset were the same as the ones used for mouse seizure detection (Table 1) with a few modifications. Firstly, we removed cross-channel metrics as these would have resulted in a very large number of features and additional computation. We also removed autocorrelation because we discovered that it was redundant when energy was used. Additionally, we split the power calculation into five frequency bands (1–4 Hz, 4.2–8 Hz, 8.2–12 Hz, 12.2–30 Hz, 30.2–55 Hz). This resulted in 12 features (*line length, root mean square, variance, standard deviation, mean absolute deviation, energy, envelope amplitude delta power, alpha power, theta power, beta power, gamma power*) per channel and hence a total of 216 features across channels. For all classification types (See Human Model Training section: intra-subject, leave-one-out, and inter-subject), highly correlated features with Pearson coefficient *r* > 0.90 were removed and the top 10, 20, 40 ranking features from ANOVA comparison with target variable were used for model training (feature IDs = 1,2,3). Additionally for intra-subject classification the top 10, 20, 40 ranking features from mutual information comparison with target variable were also used for model training (feature IDs = 4,5,6). Note: Unlike feature-sets in the mouse data, selected features were dynamically selected here and thus features could vary across folds of each classification as they were optimized for each dataset accordingly. Therefore, each feature ID might have different selected features for each fold, although selected features were the same across models for a particular fold.

### Human Model Training

The models, hyperparameter space, and fit metric used for the human dataset were the same as the ones used for the mouse dataset (Table 3 (top); DT, GNB, PAC, SGD). Models were trained and tested using three different classification schemes: 1) Models were trained and tested on the same subject (intra-subject), 2) models were trained on all subjects excluding one (leave-one-out), and 3) models were trained on some subjects and tested on other subjects (inter-subject). For intra-subject classification, we used a nested k-fold (*StratiedKfold*) strategy, where data were split into train/validation and test using 8-fold validation. Subsequently hyperparameters were tuned using a 4-fold validation of the train/validation dataset (75% training subset, 25% validation subset). For leave-one out classification, the models were trained on 23 subjects and tested on one subject for each subject (24-fold equivalent). Hyperparameters were tuned using a 4-fold validation (75% training subset, 25% validation subset). For inter-subject classification, an equivalent of 4-fold validation was used where subjects were split into 4 equal subject groups. Hence, the models were trained using 3 groups and tested on 1 group for each group. For example, for 24 subjects, the training/validation dataset consisted of 18 subjects and the testing dataset consisted of 6 subjects. For 16 subjects, as was the case when excluding subjects (Fig. 7G), the training/validation dataset consisted of 12 subjects and the testing dataset consisted of 4 subjects. As in other classification schemes, hyperparameters were tuned using a 4-fold validation (75% training subset, 25% validation subset). For Fig. 7F, subjects were separated into two clusters using a gaussian mixture model with two components and covariance_type = spherical. The PCA plot in supplementary Fig. 4B was produced by using 1/3 of the seizures from each subject, finding the max of each feature (99th percentile), and fitting a PCA to the logarithm of the extracted features (Added + 4 to eliminate values smaller than or equal to 0). For intra-subject classification, the best feature IDs were selected based on balanced accuracy (Supplementary Fig. 3A). For leave-one-out classification, the 3 feature IDs were averaged because they had a similar balanced accuracy score (Supplementary Fig. 4A). For inter-subject classification all feature IDs were used as they had similar balanced accuracy and there was only a small number of folds per model (N = 4) (Supplementary Fig. 5A).

#### Model Metrics

1. True Positive (TP): Segments that were correctly predicted as seizures.
2. True Negative (TN): Segments that were correctly predicted as non-seizures.
3. False Positive (FP): Segments that were predicted as seizures but are non-seizures.
4. False Negative (FN): Segments that were predicted as non-seizures but are seizures.
5. $\text{Recall}/\text{Sensitivity}\;=\;\frac{TP}{TP+FN}$: Proportion of identified positives.
6. $\text{Precision}\;=\;\frac{TP}{TP+FP}$: Proportion of predicted positives that were actually positive.
7. $\text{Specificity}\;=\;\frac{TN}{TN+FP}$: Proportion of identified negatives.
8.
$F1=\frac{2\times \text{Precision}\times \text{Recall}}{\text{Precision}+\text{Recall}}$

9.
$\text{BalancedAccuracy}\;=\;\frac{\text{Recall}+\text{Specificity}}{2}$

10.
$\text{SeizuresDetected}(\%)\;=\;\frac{\text{NCorrectlyDetectedSeizures}}{N\text{Seizures}}$

11.
$\text{FalseDetectionRate}\;=\;\frac{\text{NPredictedseizures}-\text{NCorrectlyDetectedSeizures}}{\text{Totalrecordingduration}(\text{Hours})}$

*Detected segments were defined as seizures if there were at least two consecutive 5 second segments.

### Statistical Tests

To compare the metric across models a one-way ANOVA was used with pairwise Tukey’s HSD multiple comparisons using the python toolbox *statsmodels*. All bar and line plots represent the mean and error-bars, or shaded regions represent the SEM. A p-value < 0.05 was considered statistically significant. All statistical tests can be found in supplementary tables 1,2, and 3.

### Code

The open-source seizure detection app, seizyML, can be found on GitHub at https://github.com/neurosimata/seizy_ml. All other code will be made available upon reasonable request.

## Results

**Figure 1:**
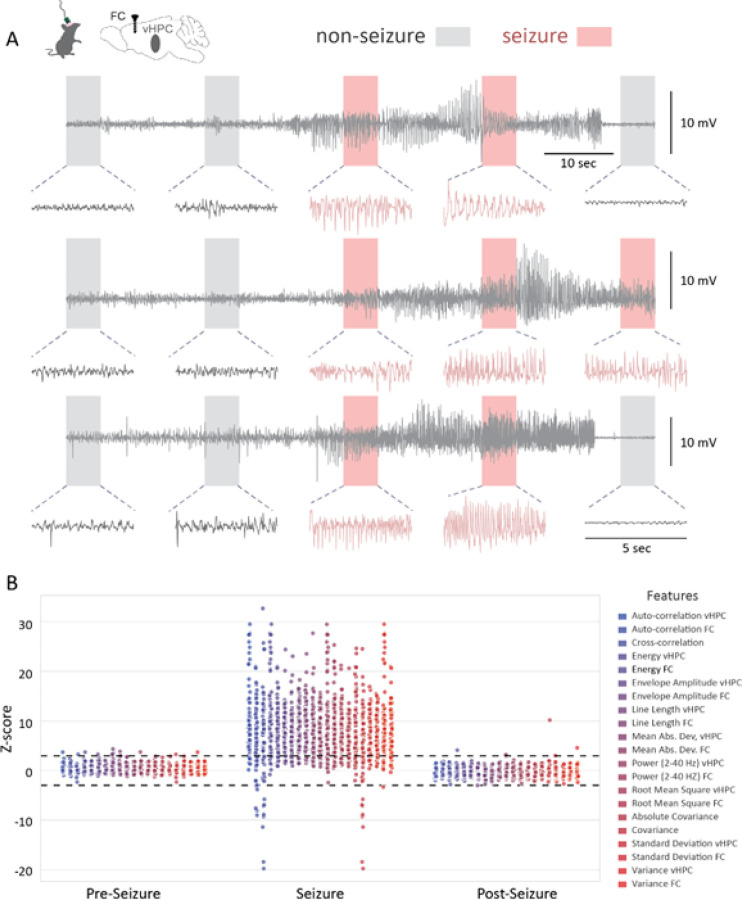
Example seizures and extracted features from the training dataset. **A)** Example seizure and non-seizure traces from ventral hippocampus (vHPC) LFP. Each row represents one seizure that was obtained from a different recording (n = 3 recordings, 2 animals), **B)** Dot plot of all extracted features before, during and after seizure from training dataset. Dotted horizontal lines indicate Z-scores of +/− 3.

An examination of even a few seizures in a well-established and reproducible model allows us to appreciate the variability and diversity of these events (Fig. 1a). This variability becomes even more apparent upon observation of the extracted features (See methods – feature selection) (Fig. 1b). Even though the extracted features robustly increase during seizure events, their variability during seizure events is greatly enhanced when compared to the periods before and after seizure events (Fig. 1b). Due to the inherent variability in seizure events, we trained machine learning models using these extracted features and examined their seizure detection performance.

**Figure 2:**
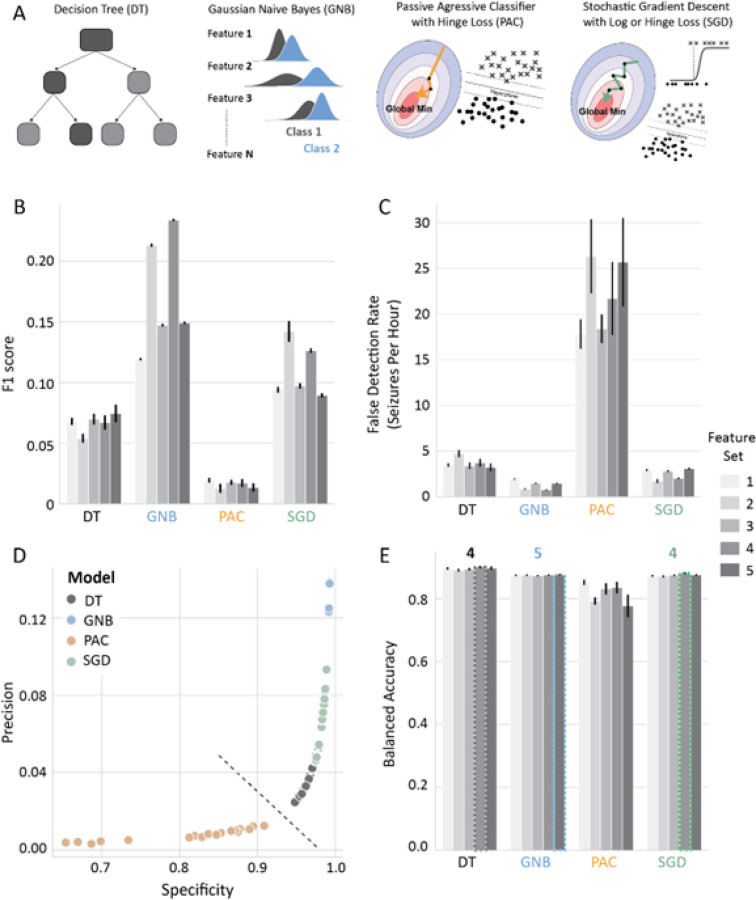
Comparison of ML models across feature-sets. **A)** Schematic of ML models compared, **B)** F1 score, **C)** False detection rate: Number of incorrectly detected seizures per hour, **D)** Scatterplot of precision vs specificity; dotted line indicates the separation of PAC with the rest of the models, **E)** Selection of feature-set from Table 2 based on balanced accuracy; Numbers on top of bars indicate the selected feature-set per model.

To achieve this, we split the data into training (11 mice, 4224 hours), and testing (15 mice, 5511 hours) datasets. We then selected 5 feature-sets (Table 1) by removing redundant features and quantifying their relevance (Table 2, See methods – feature selection). After feature selection, we chose four models (Fig. 2A) for seizure detection: decision tree (DT), gaussian naïve bayes (GNB), passive aggressive classifier (PAC), and stochastic gradient descent classifier (SGD) based on model interpretability and ability to efficiently train on our dataset (See methods – model selection). Each model was tuned (Table 3, hyperparameter selection) and then trained 5 times for each feature-set (Table 2) to account for model variability and to obtain a better estimate of their performance.

We first performed a comparison of the 4 models across all feature combinations (Fig. 2A–B). We observed that the PAC model had substantially lower F1 score (Fig. 2B) – a combined measure of model precision and recall/sensitivity (See methods – model metrics) and high false detection rate compared to the other 3 models independent of the feature combination (Fig. 2C). Additionally, the PAC trained models had lower precision and specificity across all models (Fig. 2D). Therefore, the PAC model was not considered for further analysis. For each of the three remaining models, the feature combination that resulted in the highest balanced accuracy was selected for further examination. Specifically, feature combinations 4, 5, 4 were chosen for DT, GNB, and SGD, respectively (Fig. 2E).

**Figure 3:**
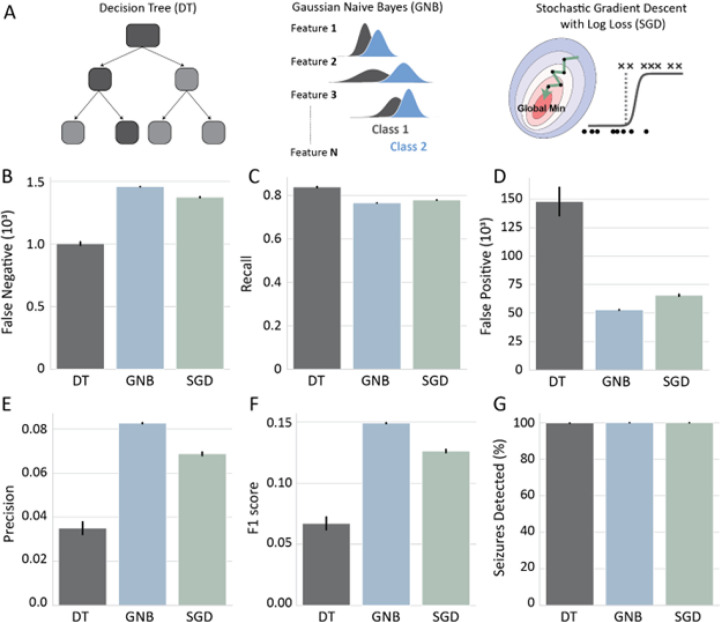
Comparison of selected ML models. **A)** Schematic of selected machine learning models based on balanced accuracy (Figure 2E). **(B-G)** Comparison of select models across metrics **B)**False Negatives, **C)** Recall, **D)** False Positives, **E)** Precision, **F)** F1 score, **G)**Percentage of seizures detected from the testing dataset.

Next, we compared the DT, GNB, and SGD models across several metrics (Fig. 3A). Overall, the DT model had the lowest number of false negatives – incorrectly classi ed seizure segments as non-seizures (Fig. 3B; DT = 147.87 ± 12.40 ×10^3^, GNB = 52.87 ± 0.16 ×10^3^, SGD = 65.69 ± 0.81 ×10^3^) resulting in the highest recall (Fig. 3C; DT = 0.84 ± 0.002, GNB = 0.77 ± 0.000, SGD = 0.78 ± 0.001) among the three models. However, it also had the highest number of false positives – incorrectly classified non-seizure segments as seizures (Fig. 3D; DT = 1.00 ± 0.013 ×10^3^, GNB = 1.46 ± 0.001 ×10^3^, SGD = 1.38 ± 0.003 ×10^3^) resulting in the lowest precision (Fig. 3E; DT = 0.03 ± 0.003, GNB = 0.08 ± 0.000, SGD = 0.07 ± 0.001). These results indicate that the DT model was the most sensitive and the least precise among the three models. On the other hand, the GNB model was the most precise (Fig. 3E) and had the highest F1 score (Fig. 3F; DT = 0.07 ± 0.005, GNB = 0.15 ± 0.000, SGD = 0.13 ± 0.001). The SGD model had an intermediate performance overall with a lower F1 score than the GNB model (Fig. 3F). Even though the DT model has the highest recall, it had a similar if not slightly worse performance at seizure detection (See methods – model metrics) than GNB and SGD models which detected all seizures in the test dataset (Fig. 3G; DT = 99.80 ± 0.033%, GNB = 100.00 ± 0.000%, 100.00 ± 0.000). These results demonstrate that simple and interpretable machine learning models can be very efficient for seizure detection but vary in their reliability and prediction accuracy.

**Figure 4:**
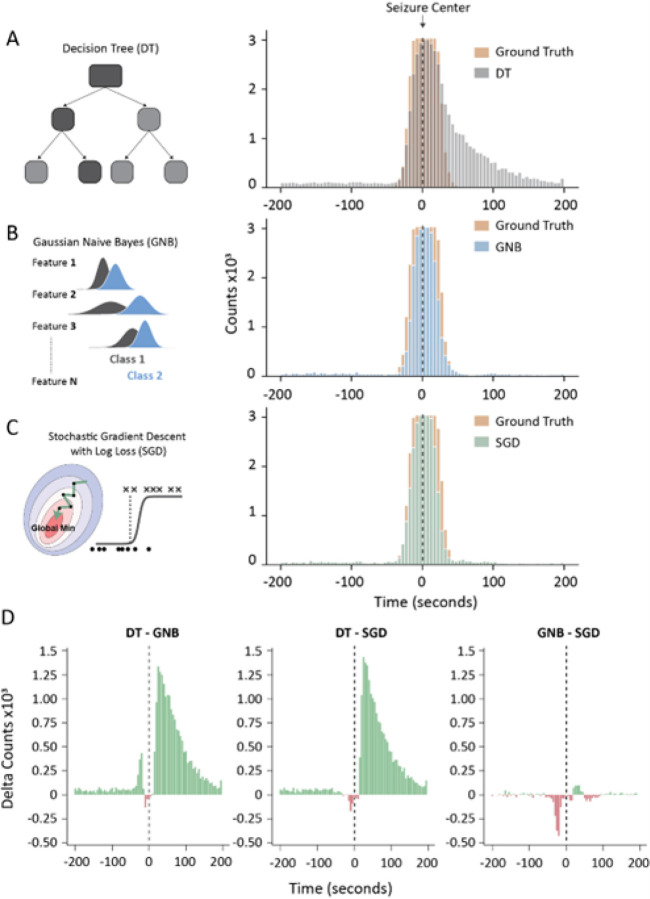
Seizure prediction across time vs ground truth data. **(A-C)** Number of ground truth vs predicted seizure across time bins. **A)**DT, **B)** GNB, **C)** SGD. **D)** Difference between model predictions across the 3 pairs. Dotted line represents the seizure center.

Even though the DT model had significantly higher recall than the GNB model, the number of seizures detected was not superior (DT: 99.80 ± 0.03%, GNB: 100.00 ± 0.00%). Given that the recall of all three models was lower than the proportion of seizures detected, we investigated how the predicted seizure bins across time compared between the three models and ground truth data. When comparing the predicted seizure bins to ground truth data, we observed that the models detected the center of the seizure with higher accuracy than seizure boundaries (Fig. 4A–C). This is not surprising given that the features that were used to train these models do not increase as robustly at the designated seizure boundaries (Supplementary Fig. 1). This observation could explain why the proportion of segments predicted correctly as seizures is lower than the proportion of detected seizuresD. Interestingly, it seems that the increased recall of the DT model arises from high detection of seizure offset segments.

However, the DT model dramatically misclassifies seizure offset (Fig. 4A, D), which likely accounts for its decreased precision. This was not specific to the selected feature-set chosen to train the DT model or the depth of the tree, as all DT models tested here overestimated seizure offset predictions (Supplementary Fig. 2).

**Figure 5:**
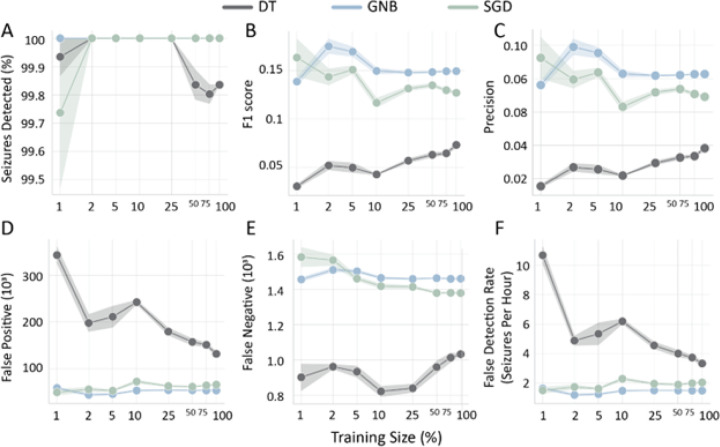
Model performance as a function of training data size. Performance of ML models across key metrics for increasing portions of the training dataset. **A)** Percent seizures detected, **B)** F1 score, **C)** Precision, **D)** False Positives, **E)**False Negatives, **F)** False detection rate: Number of incorrectly detected seizures per hour.

Manual inspection of EEG datasets to create training labels is costly and laborious. To examine the dataset size required to achieve good model performance and seizure detection, we trained models on increasing size of data portions (Fig. 5). The GNB model detected all seizure events from just 1% of the training data even though its performance based on F1 score and balanced accuracy, seems to stabilize at 10% of the training data (Fig. 5A–B). The SGD model detected 99.74% of all seizures at 1% of training data and detected all seizures at 2.5% of the training data whereas its F1 score seems to have stabilized around 10% of the training data, although consistently below the GNB model (Fig. 5A–B). The DT model detected 99.93% of all seizures at 1% of training data and detected all seizures at 2.5% of the training data, but its detection dropped to 99.84% at 100% of training data size (Fig. 5A). This likely resulted from a DT model optimization to increase precision and reduce false positives (Fig. 5C–D), resulting in higher number of false negatives (Fig. 5E). In addition, the F1 score of the DT model kept improving as the training data size increased up to the full dataset. However, the F1 score, and false detection rate (Fig. 5D, F), were lower than the GNB and SGD models across training data sizes. As observed before, the DT model had a much lower number of false negatives in comparison to the SGD and GNB models even though it had a reduced seizure detection overall (Fig. 5A, E). Therefore, the GNB and SGD model perform well even with smaller training data sizes and quickly achieve a stable performance. On the other hand, the DT model requires a large amount of data to improve its precision at the cost of reduced seizure detection.

**Figure 6:**
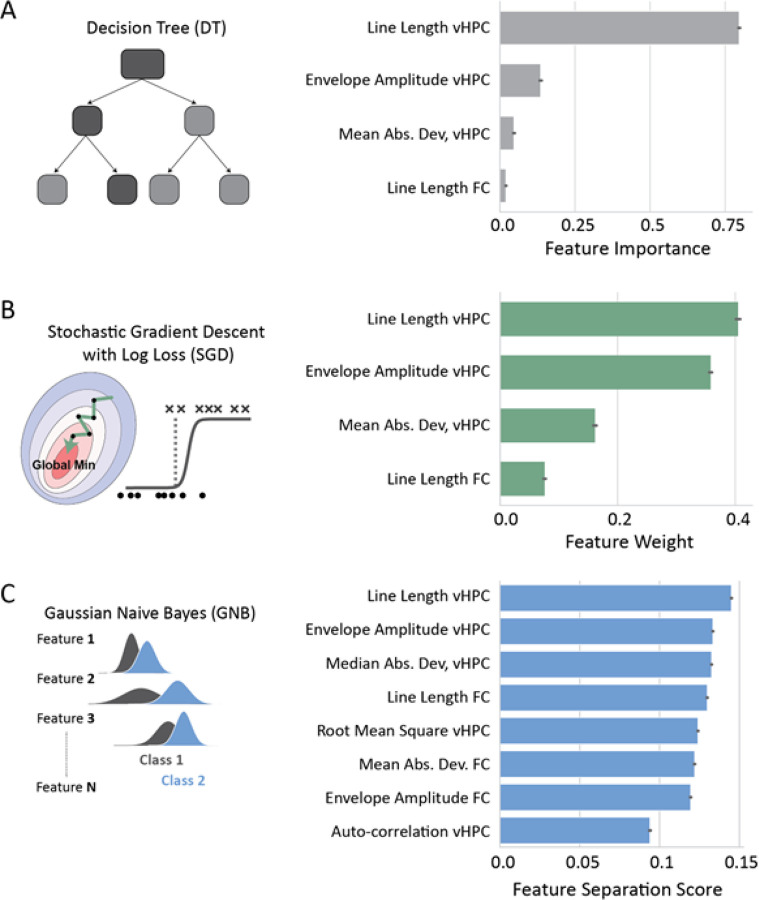
Feature importance for classification. **A)** DT: Feature importance, **B)** SGD: Feature weight, **C)** GNB: Feature separation score. Each feature-set has a cumulative sum of 1.

To further understand how these models classified EEG segments we extracted metrics which quantified the influence of each feature on model predictions (See methods: feature contributions; DT: feature importance, SGD: feature weight, GNB: feature separation score). This analysis revealed that in the DT model the *line length of the vHPC* was by far the most important feature with a value of 0.80. The second most important feature was the *envelope amplitude of the vHPC* with only a value of 0.14, while the two other features had negligible importance, each scoring less than 0.05 (Fig. 6A). In contrast the SGD model had a more balanced weight across features, with the *line length of the vHPC* also having the highest weight score of 0.40. The envelope amplitude of the vHPC was a close second, with a feature weight of 0.36, while the other two features had a combined score of 0.24 (Fig. 6B). Lastly, the GNB model does not have an in-built metric for feature importance thus we calculated a feature separation score based on the distribution of each feature from the trained GNB models (See methods – feature contributions). Our analysis indicated that most features had comparable scores, although the *line length of vHPC* had a marginally higher score (Fig. 6C). Thus, the GNB model appears to have a more balanced feature contribution for its predictions. Overall, this analysis reveals that the *line length of vHPC* feature is a key contributor to seizure detection in this dataset, whereas feature contributions varied across models. Intriguingly, the models with more balanced feature contributions also had a superior performance.

Finally, to test the validity of these models on multi-channel datasets from human EEG recordings we utilized the Children’s Boston Hospital MIT dataset (CHB-MIT) (Shoeb & Guttag, 2010) as it has been extensively used for benchmarking ML models (Siddiqui et al., 2020). This dataset consists of 24 recordings from 23 human subjects, where each recording has 23–26 channels. We selected 18 channels that were common across subjects, divided the data into 5 second bins, and extracted features (See methods: Human Data and Human Feature Selection).

**Figure 7:**
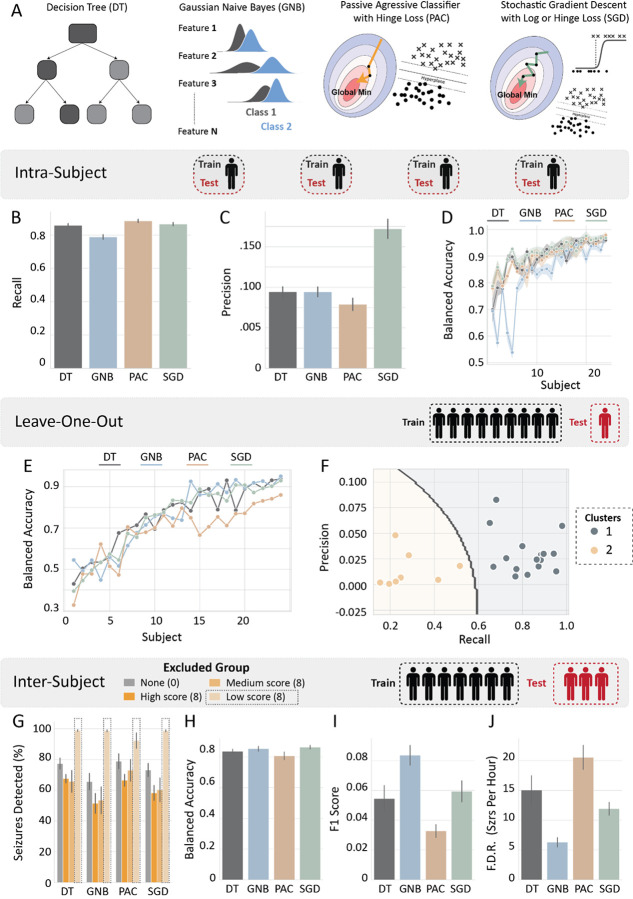
Performance of ML models in the Boston Children Hospital MIT (CHB-MIT) EEG dataset. **A)** Schematics of machine learning models used for classification. **(B-D)** Model metrics for intra- subject classification: **B)** Recall, **C)** Precision, **D)** Balanced accuracy across subjects (ordered by ascending score). (**E-F)** Model metrics for leave-one-out subject classification: **E)** Balanced accuracy across subjects (ordered by ascending score), **F)** Clustered subjects based on precision and recall scores using a gaussian mixture model (grey line indicates decision boundary). **G)** Percent Seizures detected across models and excluded groups for inter-subject classification. **(H-I)** Model metrics for inter-subject classification when the 8 subjects that had the worst score were excluded: **H)**Balanced accuracy, **I)** F1 score, **J)** False detection rate (seizures per hour). Note that subject numbers in **D)** and **E)** correspond to the rank of the balanced accuracy scores that the subjects received in each classification paradigm and may not match between classification scores. Subject ID was intentionally excluded.

We first used 8-fold cross-validation to assess the performance of models that were trained and tested on the same patient (intra-subject classification). All models achieved similar recall scores that were above 0.86 (DT: 0.86 ± 0.011, PAC: 0.88 ± 0.010, SGD: 0.87 ± 0.009) with the GNB model reaching a slightly lower score of 0.79 ± 0.014 (Fig. 7A). Interestingly, the SGD model achieved a much better precision score than all other models (SGD: 0.17 ± 0.012 vs DT: 0.09 ± 0.006, PAC: 0.08 ± 0.008, GNB: 0.09 ± 0.006) (Fig. 7B). Indeed, across scores the SGD model achieved the best performance, whereas the GNB and PAC models had the worst performance for intra-subject classification (Supplementary Fig. 3). As expected, we observed that there was some variability in the classifications scores between subjects (Fig. 7C).

To further examine how the model predictions generalize between subjects, we trained models across all subjects and excluded 1 subject for testing (leave-one-out classification). We found that model performance substantially varied across different subjects (Fig. 7E) and found that subjects fell into two groups when clustered by recall and precision (Fig. 7F). Moreover, the seizure features of low scoring subjects (cluster 2 from Fig. 7F) were similar as indicated by a PCA plot (Supplementary Fig. 4B). These findings indicate that subjects from the low-score group did not exhibit robust alterations of EEG waveforms during seizures. This can also be illustrated from example traces of seizures from subjects of the two groups (Supplementary Fig. 4C).

Finally, to test how well the models can detect whole seizure events instead of seizure segments, models were trained on 75% of the subjects and tested on 25% of the subjects (inter-subject classification; See methods: Human Model Training). Additionally, we trained models where we excluded subjects with low, medium, and high scores (average of recall and precision; N = 8 excluded subjects per group) and examined their performance. We found that exclusion of subjects with high or medium scores had little effect on seizure detection when compared to models trained on the full dataset (Fig. 7G). However, exclusion of subjects with low scores dramatically increased seizure detection to 98% across all models (DT: 98.86 ± 0.613, GNB: 98.68 ± 0.687, SGD: 98.68 ± 0.687) besides PAC that only reached a detection rate of 92.30 ± 5.076% (Fig. 7G). Indeed, the PAC model had the lowest F1 score and highest false detection rate, whereas the GNB model had the highest F1 score and lowest false detection rate, with the DT and SGD models falling somewhere in the middle (Fig. 7H–J; Supplementary Fig. 5). Overall, these data suggest that interpretable ML models can reliably detect electrographic seizures from multi-channel human EEG recordings with high sensitivity.

**Figure 8:**
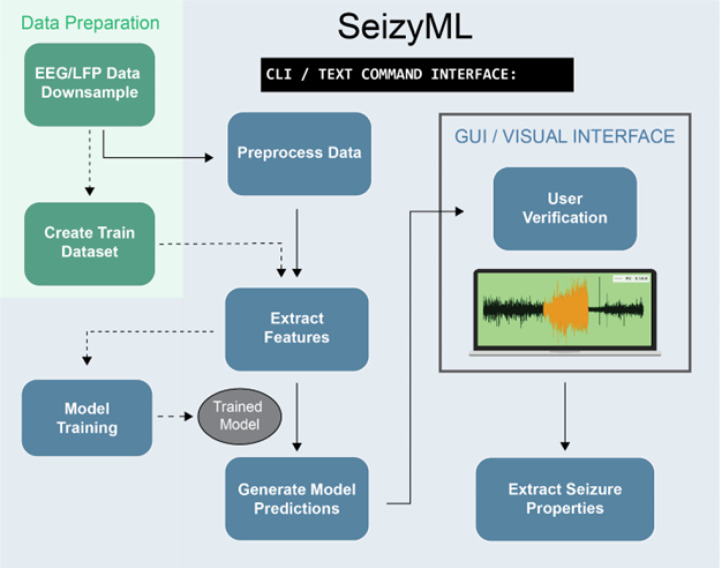
SeizyML pipeline. Green area denotes data preparation steps that need to be performed by the user before the data are ready for processing by SeizyML. Blue area highlights the main processing steps of the SeizyML application.

Here we observed that interpretable ML models with simple feature extraction were very effective at detecting seizures from a well-established model of chronic epilepsy in mice (Basu et al., 2022). To couple the high model sensitivity with enhanced accuracy we created an open-source application for semi-automated seizure detection, SeizyML, that combines model predictions with manual curation of the detected seizure events. The outline of the pipeline is illustrated in Fig. 8. Before the app can be used, the raw LFP/EEG data should be downsampled (100 Hz, 5 second windows) and must be converted from their native format (depending on recording apparatus) to HDF5. A small training dataset needs to also be prepared to train and calibrate the model. Then using the command line interface of SeizyML, the data are preprocessed, features are extracted, and model predictions are generated. Following that a simple GUI allows the user to accept or reject the detected seizures. Lastly, seizure properties can be extracted from the detected seizures using the seizyML CLI. Importantly, the app can be easily extended to use any machine learning (ML) model, channel number and features. Although, care should be taken since some ML models cannot perform well on big datasets especially on large number of features (including decision tree models used here).

## Discussion

Here we have created an open-source python application, SeizyML, for semi-automated seizure detection using ML models. We propose that a semi-automated approach that combines fast and sensitive machine learning models with seizure verification from human operators is a crucial first step in the automation of seizure detection. Allowing operators to understand model decisions and verify detected seizures can enhance their trust of the underlying model and will also help to understand how to further improve ML models.

We compared the performance of four linear models for automated seizure detection: gaussian naïve bayes (GNB), decision tree (DT), stochastic gradient descent classifier (SGD) and passive aggressive classifier (PAC). Even though most models detected all seizures in our mouse test dataset (608 seizures), we found that the GNB model had the best F1 score (Fig. 2), had simple tuning (only one hyperparameter), needed only a small amount of training data to reach good performance and it is highly interpretable. Furthermore, it had the best F1 score across models when tested on inter-subject classification of a human multi-channel EEG dataset (Fig. 7). Our model comparisons further revealed that the PAC model had the worst performance across multiple metrics in both mouse and human datasets (Figs. 2 & 7). It’s possible that the suboptimal performance of the PAC model arises from the aggressive optimization algorithm that misses the global minimum. This is supported by the fact that the SGD classifier with a different optimization algorithm, but the same loss function (Table 3) outperformed the PAC model (Fig. 2). The DT model had the highest recall and lowest precision when compared to DT and SGD models in our mouse dataset (Fig. 3). However, the high recall of the DT model did not translate to better seizure prediction (Fig. 2). Both the high recall of the model and low precision seems to arise from misclassifications of the seizure termination (Fig. 4) and the poor performance of the model could be attributed to it heavily relying on one feature (Fig. 6). The SGD model had comparable performance to the GNB model although it had a lower F1 score (Figs. 3 & 7). Additionally, the SGD model has many hyperparameters and took the longest to tune using grid search making it more time-consuming to train. Nevertheless, the SGD model outperformed all other models in human intra-subject classification. Therefore, it would be useful to compare the performance of both models on a dataset before selecting the model. For this reason, we have included both the SGD and GNB models in our SeizyML pipeline, where the best model and feature set will be selected based on the training data.

### Limitations and Future Directions

This study aimed at finding simple and interpretable ML models for offline seizure detection. Our method using 5 second windows is not able to detect the seizure onset with high temporal precision. If detecting seizure onset/offset is of primary interest, our model could be combined with additional time-sensitive algorithms such as wavelet transform or Empirical mode decomposition approaches (Chen et al., 2017; Molnár et al., 2023). Although, these algorithms tend to be computationally expensive and thus it would be inefficient to be used in isolation. Furthermore, we downsampled the LFP/EEG data to 100Hz and excluded high frequency components that could be important for seizure detection (Bragin et al., 2004; Molnár et al., 2023). In addition, we did not perform an exhaustive search of interpretable ML models due to time constraints. However, this approach was sufficient to detect all seizures in our mouse dataset and has also resulted in a fast and efficient ML pipeline.

One crucial consideration is that most automated seizure detection algorithms, including the ones here, are often restricted in classifications of non-seizure vs seizure periods. Yet, there is a broad range of seizures as categorized by clinical outcomes (such as focal, generalized tonic-clonic, etc.) (Fisher et al., 2017; McCallan et al., 2023). Detecting these different types of seizures is crucial in epilepsy as it can assist in enhanced diagnosis, more targeted therapies, and better control of seizures (McCallan et al., 2023). However, supervised approaches can only be as good as our definitions of these seizure types. For example, here we have shown that subjects from the CHB-MIT dataset fell into two groups based on their classification scores and features. Importantly, in cases where EEG seizure pro les are not robust, other sensors such as accelerometers, EMG, and EKG could be used in conjunction to help improve the reliability and probability of seizure detection. Additionally, it would be useful to help develop interpretable deep learning models that could further improve seizure detection and automate feature extraction across different epilepsy types.

Moreover, even though definitions of seizure types are improving, they are based exclusively on clinical symptoms (Fisher et al., 2017), and do not take into account the pathophysiology of neuronal networks and the mechanism of seizure generation. Therefore, improving seizure definitions based on the pathophysiology of neuronal networks is of paramount importance. We believe that creation of diverse seizure datasets (from multiple brain regions, recording systems, seizure types) with improved seizure definitions should be of utmost priority in order to improve epilepsy diagnosis and train better ML models for automation of seizure detection and could potentially be used to classify epilepsy subtypes.

## Data Availability

The datasets used and/or analysed during the current study will be made available from the corresponding author on reasonable request.
